# Assessing the Potential Distribution of Asian Gypsy Moth in Canada: A Comparison of Two Methodological Approaches

**DOI:** 10.1038/s41598-019-57020-7

**Published:** 2020-01-08

**Authors:** Vivek Srivastava, Verena C. Griess, Melody A. Keena

**Affiliations:** 1 University of British Columbia, Faculty of Forestry, Department of Forest Resources Management, Vancouver, V6T1Z4 Canada; 2 Northern Research Station, USDA Forest Service, Hamden, CT 06514 United States

**Keywords:** Forestry, Invasive species

## Abstract

Gypsy moth (*Lymantria dispar* L.) is one of the world’s worst hardwood defoliating invasive alien species. It is currently spreading across North America, damaging forest ecosystems and posing a significant economic threat. Two subspecies *L*. *d*. *asiatica* and *L*. *d*. *japonica*, collectively referred to as Asian gypsy moth (AGM) are of special concern as they have traits that make them better invaders than their European counterpart (e.g. flight capability of females). We assessed the potential distribution of AGM in Canada using two presence-only species distribution models, Maximum Entropy (MaxEnt) and Genetic Algorithm for Rule-set Prediction (GARP). In addition, we mapped AGM potential future distribution under two climate change scenarios (A1B and A2) while implementing dispersal constraints using the cellular automation model MigClim. MaxEnt had higher AUC, pAUC and sensitivity scores (0.82/1.40/1.00) when compared to GARP (0.70/1.26/0.9), indicating better discrimination of suitable versus unsuitable areas for AGM. The models indicated that suitable conditions for AGM were present in the provinces of British Columbia, Ontario, Quebec, Nova Scotia and New Brunswick. The human influence index was the variable found to contribute the most in predicting the distribution of AGM. These model results can be used to identify areas at risk for this pest, to inform strategic and tactical pest management decisions.

## Introduction

Forest Invasive Alien Species (FIAS) are of serious concern to biodiversity around the world and their impacts on native plant species, communities, and on associated environments are widely recognized to have both economic and ecological impacts^1,2^. In Canada, over 80 Forest Invasive Alien Species (FIAS) have been introduced since 1882, most notably Dutch elm disease, white pine blister rust, rusty tussock moth, Asian longhorned beetle, emerald ash borer and European gypsy moth^3^. It is estimated that these FIAS destroy roughly 400,000 hectares (ha) of forest every year^4^. The establishment of FIAS has continued to rise^5,6^, and their impacts are anticipated to be substantial throughout all environments^7,8^. Moreover, the global, national, and regional spatial patterns of FIAS invasions in the future are likely to be different due to technological advances in transportation and international trade^9^, increase in human population^10^, and climate change^11^. Gypsy moth (*Lymantria dispar* L.) is listed as one of the 100 worst invasive alien species in the world, selected from global invasive species database by the International Union for Conservation of Nature (IUCN). Gypsy moth can cause serious defoliation and weakening of trees and shrubs, that either leads to tree death directly or indirectly by subsequent infestation by a secondary pest. Currently, gypsy moth is spreading in North America (primarily in the United States) damaging both commercial and native forest ecosystems over much of the introduced range. The pest also poses a significant economic threat to several other countries that are taking actions to prevent its introduction^12^. Two subspecies, Asian gypsy moth (*L*. *dispar asiatica* Vinkovskij), distributed throughout temperate Asia, and the Japanese gypsy moth (*L*. *dispar japonica* Motschulsky), distributed throughout Japan, are of serious concern although they have not yet permanently established (though several incursions have been eradicated) outside their native range^13,14^. The two subspecies, *L*. *d*. *asiatica* and *L*. *d*. *japonica* here after collectively referred to as Asian gypsy moth (AGM) have a broad host range (over 600 plant species, including conifers) and the females are flight capable^15^. They have an affinity to fly towards light sources and strong dispersal traits, which makes them a greater threat than their European counterpart. The AGM females have been found to fly to lights in port areas and lay their egg masses on cargo and the superstructure of ships. A study by Paini *et al*.^13,14^ found that more than 7,500 ships that have the potential to carry AGM egg masses from Asia arrived in 2005 at Canadian ports that fall into the climatically suitable range of AGM. Several detections of AGM egg masses on vessels coming from Asian ports have occurred in Canada (the first eradication effort occurred around the Vancouver port in 1992^16^) which have led to the implementation of international phytosanitary measures to prevent AGM establishment that are significantly reducing the risk of invasion. Because there is a continuing risk of introduction, it is important to predict potential distribution and factors that influence their ability to establish^17^.

Species distribution models (SDMs) are commonly used to provide information on potential distribution range of FIAS, which in turn is critical for conservation and management planning and for understanding FIAS ecology^18^. SDMs build on the general concept of a fundamental niche and forecasted species distributions that are dependent on the underlying modelling algorithm that is applied^19^. SDMs are trained with species occurrence data and associated environmental layers from one range, and then projected onto a different range in order to identify regions with relative environmental suitability for a given FIAS. The theory behind this method is the similarity between native and non-native regions of the FIAS^20^. In the past, researchers have used both correlative (GARP) and partially-mechanistic (CLIMEX) approaches to predict the global potential distribution range of AGM^13,14^. Peterson *et al*.^14^ used the Genetic Algorithm for Rule-set Prediction (GARP) whereas Paini *et al*.^13^ used CLIMEX to map global climate suitability for AGM. These studies looked at global species distributions of AGM with no study focused on a Canada-wide scale. We therefore aimed to compare two SDM approaches, MaxEnt and GARP, to check if they provide reliable distribution estimates in AGMs native range and realistic potential distribution maps for AGM in Canada. MaxEnt and GARP are capable of providing robust predictions with presence-only data and have often outperformed classical modeling approaches, such as domain, bioclim, and logistic regression^21^. Only a few studies have reported mixed performances of GARP and MaxEnt with respect to their predictive success in unsampled regions^22,23^. To support improved future surveillance of AGM, we also investigated the null hypothesis that there would be no difference in the potential habitat of AGM under changing climates. To do this we used the two SDMs (MaxEnt and GARP) we had developed and mapped potential distribution of AGM in Canada under two climate change scenarios A1B and A2. Basically, the A1B scenario is characterized by a very rapid economic growth, low population growth and rapid use of more efficient technology whereas, the A2 scenario refers to a heterogeneous world with high population growth and less concern for economic development^24^.

## Results

### Potential distribution of AGM

**Figure 1:**
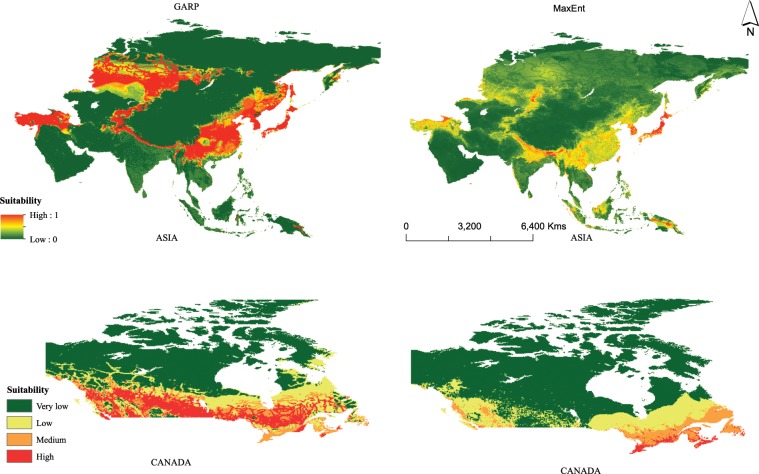
Predicted potential distribution of AGM in Asia and Canada, using GARP and MaxEnt. For Asia, higher probability (red colors) represent areas suitable for AGM. Zero probability or lower probability (dark green) indicates areas less suitable. Whereas, for Canada the continuous suitability values (0–1) from GARP and MaxEnt outputs were classified as: very low (0–0.25), low (0.25–0.50), medium (0.50–0.75) and high (0.75–1.00) for easy interpretation and comparison purposes.

Modelling results for AGM distribution from GARP and MaxEnt showed a similar distributional pattern in the species’ native range. However, different suitability scores were obtained from both SDMs. GARP assigned larger areas a high suitability score than MaxEnt (Fig. 1). Areas in which modelling results are in agreement, include Japan, North and South Korea, central and western parts of Russia, eastern and southern China, Indo-Himalayan range, Laos, Parts of Indonesia and Papua New Guinea, Kyrgyzstan, Tajikistan, Northern Afghanistan and Kazakhstan, Turkey and the north-western regions of Iran. Additionally, suitability was considered high in southern parts of India and Sri Lanka. These predictions are well in agreement with the occurrence records found in the literature^25^.

**Figure 2:**
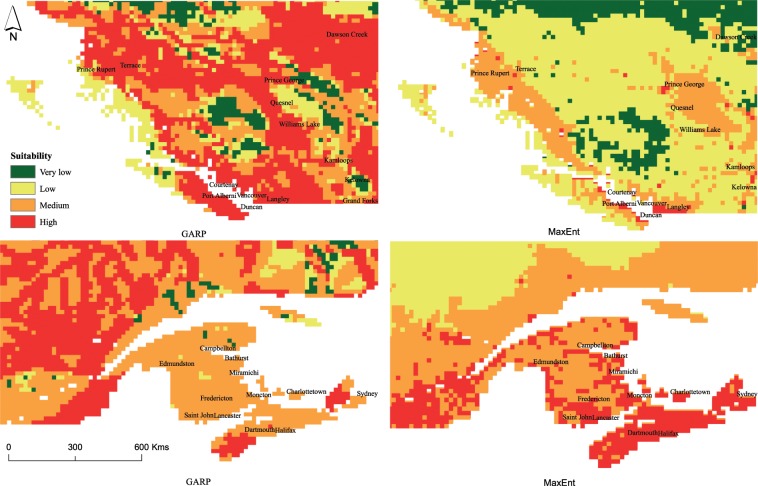
Potential distribution (enlarged view) of AGM using GARP and MaxEnt in Canada at 20 km^2^ spatial resolution.

Predictions obtained from transferring these models to nonnative range (in Canada) using both approaches however, did show substantial differences (Figs. 1 and 2).

Areas modelled to be highly suitable using GARP were found in British Columbia (BC), Alberta, Saskatchewan, southern regions of Manitoba and Ontario (excluding Toronto) and parts of Quebec, Nova Scotia, Prince Edward Island and Newfoundland. MaxEnt results identified coastal BC, areas around cities of Vancouver and Toronto, Southern Quebec, Prince Edward Island, Nova Scotia and southern parts of New Brunswick and Newfoundland to be of high suitability. Differences between the distribution predictions was recorded for all suitability classes across Canada, with major differences observed in Manitoba, Saskatchewan and Alberta (Figs. 1 and 2). Similar patterns of predicting higher suitability scores for larger regions was recorded from GARP across the entire nonnative range.

### Environmental responses, variable contribution and model evaluation

**Figure 3:**
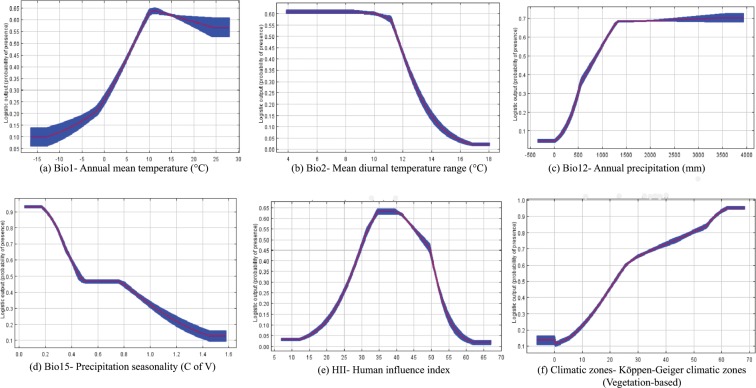
Relationships between environmental predictors and the probability of the presence of AGM: Red curves show the mean response and blue bands around them are ±1 standard deviation calculated using 10 independent data subsets.

The occurrence data and modelled distributions indicate that areas receiving annual precipitation between 800 and 3,800 mm and a mean annual temperature range of 5 to 25 °C are suitable for AGM establishment. These areas have HII index value above 25 (Fig. 3) and cover a large range of climatic zones (Table 1).

**Table 1:** Climatic regions according to the Köppen-Geiger climate classification system at locations where AGM was found.

Climate type	Description	Location
Cfb	Temperate oceanic climate (temperate, without dry season, warm summer)	Inner Mongolia, China
Cfa	Humid subtropical climate (temperate, without dry season, hot summers)	Guangxi, China
Cwa	Monsoon-influenced humid subtropical climate (temperate, dry winter, hot summer)	Nantou County, Taiwan
Dwa	Monsoon-influenced hot-summer humid continental climate (continental, dry winter, hot summer)	Gyeongsangnam-do, South Korea
Dfa	Hot-summer humid continental climate (Continental, without dry season, hot summer)	Jeollabuk-do, South Korea
Dfb	Warm-summer humid continental climate (Continental, without dry season, warm summer)	Nagano Prefecture, Japan
Dfc	Subarctic climate (Continental, without dry season, cold summer)	Irkutsk Oblast, Russia
Dwc	Monsoon-influenced subarctic climate (Continental, dry winter, cold summer)	Irkutsk Oblast, Russia
BSk	Cold semi-arid climate (Arid, steppe, cold)	Inner Mongolia, China
Dwb	Monsoon-influenced warm-summer humid continental climate (Continental, dry winter, warm summer)	Gansu, China

**Figure 4:**
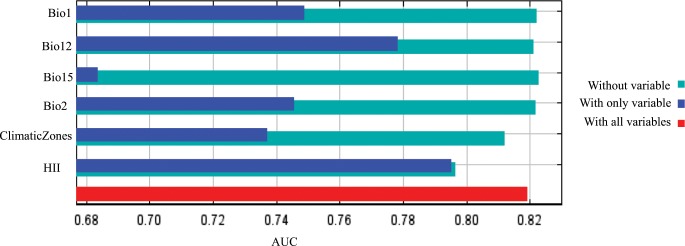
Jackknife test for AUC of environmental variable importance for the MaxEnt model.

The jackknife test identified the Human Influence Index (HII) and annual precipitation (Bio 12) as the most important predictors of AGM distribution (Fig. 4). HII made the largest contribution to the MaxEnt model of AGM distribution when used in isolation and reduced the model’s predictive ability the most when omitted. HII, annual precipitation and seasonality of precipitation were the strongest predictors of AGM potential range contributing 46.5, 26.2 and 17.8% respectively, showing that in addition to precipitation, anthropogenic factors can have a huge impact on the distribution of this species.

The AUC, pAUC and sensitivity scores for MaxEnt and GARP models were higher than 0.7, 1 and 0.8 suggesting that both of the approaches had good predictive capability (interpolation). However MaxEnt had higher AUC, pAUC and sensitivity scores (0.82, 1.40 and 1) compared to GARP (0.70, 1.26 and 0.9) indicating better discrimination of suitable versus unsuitable areas for AGM.

### Dispersal mapping under two climate change scenarios

**Figure 5:**
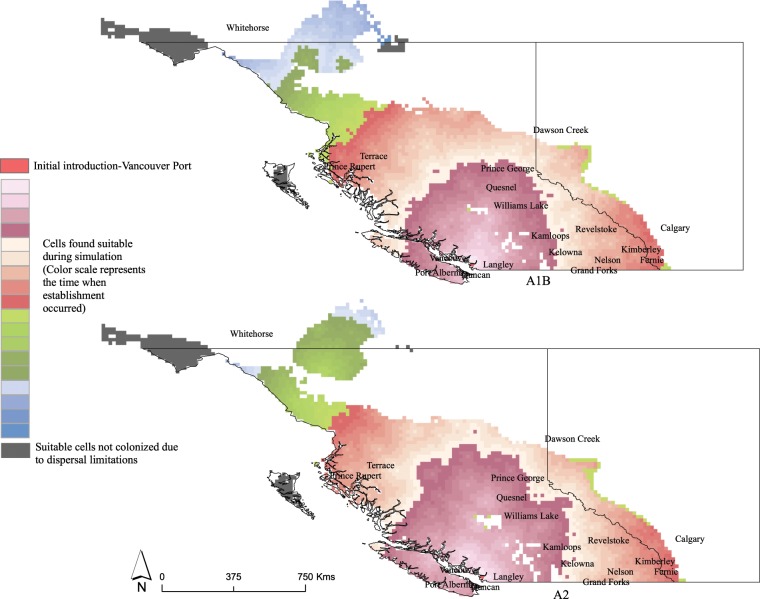
Dispersal restricted future distribution of AGM under A1B and A2 climate change scenarios. Color gradient from rose to dark red represents first 10 years of the simulation timeframe when colonization first occurred, the light sand to cherry red color gradient represents the next 10 years followed by green and sky blue color gradients (years 2031–2050). Red pixel indicates the hypothesized point of AGM introduction while the dark grey pixels represent suitable areas that were not colonized due to dispersal limitations.

MigClim simulations show that at the end of simulation run (i.e. the potential distribution of AGM under specified dispersal restrictions) a total of 16,450 cells were colonized in the A1B scenario, whereas 16,654 cells were colonized under A2 scenario (Fig. 5).

In the first 10 years of the modelling timeframe, areas in coastal BC, around Vancouver, Duncan, Port Alberni, Courtenay, Quesnel and Williams Lake are likely going to provide suitable habitat to AGM. In next 10 year period (years 2021–2030) areas east of Kamloops, Kelowna and Prince George will provide suitable habitat for AGM dispersal and establishment. Areas around Prince Rupert and Terrace may also be suitable in the same period. Under the A2 climate change scenario the potential area colonized during the second and third 10 year timeframes would be larger compared to A1 scenario. In 2031–2050, areas south of Whitehorse near Teslin and Juneau, as well as areas around Watson Lake would be expected to be colonized. Areas around Graham Island and Moresby Island including Naikoon Provincial Park (PP) and Gwaii Haanas National Park (NP) and NP situated southwest to Whitehorse (Kluane) and Tatshenshini-Alsek PP may not get colonized due to dispersal limitations.

## Discussion and Conclusions

Both modelling approaches predicted large areas suitable for AGM in Canada. These findings are similar to studies conducted in the past by Peterson *et al*.^14^ and Paini *et al*.^13^. GARP and MaxEnt performed well for AGM’s native range with relatively high AUC, pAUC and sensitivity scores. However MaxEnt out performed GARP in determining potential distributions of AGM in the native range. This finding is in line with other studies that found MaxEnt outperforming alternative approaches^18,26^. We found GARP to overestimate suitable areas in both native (e.g. Russia and Indo-Himalayan range) and nonnative range of AGM (e.g. Yukon, Nunavut, Alberta and Saskatchewan). In contrast MaxEnt predictions were close to the known distributional range of AGM in the native range. Since AGM is not currently established in Canada, we did not have any data to test transferability success of the approaches. As a surrogate for AGM we compared the predictions from both models by overlaying interception location records of EGM (European gypsy moth) provided by the CFIA (collected as a part of annual pest survey), since EGM and AGM are assumed to have similar ecological characteristics and hosts^27,28^. We found that MaxEnt predictions matched more closely to EGM distributions in Canada than GARP. The better performance of MaxEnt can be attributed to its ability to fit complex functions including interactions amongst predictors and use of penalty functions to avoid overfitting^29^. GARP predictions are often criticized for overestimating the distribution range^26^ as it tends to omit less important relationships in the data (temperature, in our case)^30^.

Our results indicated that the total suitable area for AGM will increase in the A2 scenario, which led to a rejection of our original hypothesis –that there would be no difference in the potential habitat of AGM under a changing climate. Climate models have projected that by 2100, eastern Canada will be approximately 3–5 °C warmer, with increased winter precipitation^31^, this rise in temperature which could eventually lead to much higher probabilities of AGM invasions. Suitable conditions for AGM were modelled in areas with annual temperatures between 5 and 25 °C, but the optimum range (probability of presence >0.60, based on maximum test sensitivity and specificity threshold^32^) was between 11–25 °C. This optimum range is in agreement with the recent findings of Limbu *et al*.^12^ that AGM populations may struggle in regions that experience longer periods of temperatures ≥30 °C and that AGM survival was highest between 15 and 25 °C. Canadian areas classified as suitable were located within the Koppen-Geiger climatic zones- DFb (average temperatures below 22 °C) and DFc, which represents subarctic climate where 1–3 months have an average temperature above 10 °C. We included an additional variable HII to account for dispersal and human footprint and found that it contributed the most to determining the distribution of AGM. Areas predicted to be suitable had HII values above 25. This backs the notion that FIAS distributions are heavily impacted by the influence of humans on the landscape^33^ and that human activity can significantly affect the distribution of FIAS.

The AGM distribution maps generated by our models were different from those produced by Peterson *et al*.^14^ and Paini *et al*.^13^, who found large areas in Alberta, Saskatchewan and Manitoba to be suitable while our MaxEnt model showed only limited suitability in these provinces. This could possibly be due to the following limitations of these studies: (1) GARP has been criticized for over predicting distribution ranges^18^ and failure to model less important relationships in the data^30^. Additionally GARP has been also found to be less accurate than MaxEnt in several studies^34^. (2) Bioclimatic variables included in the SDMs by Peterson *et al*.^14^ did not account for anthropogenic impacts on the response variable (probability of species occurrence). (3) The authors have ignored the dispersal limitations of the FIAS in the modelled distributions. This is despite the fact that FIAS invasions increase as dispersal capabilities increase, often aided by human activities^35^. (4) It is important to constrain the pseudo-absence (PA) locations to the same geographic range as presences for accurate predictions^36^, however this approach was not followed by Peterson *et al*.^14^ while selecting PA samples.

These findings suggest that pest surveillance in these provinces may need to be reevaluated. These spatial uncertainties of the individual models can be minimized by adopting a machine learning approach based on combining multiple models, but this does have limitations. The ensemble approach may misinterpret suitability if individual model predictions overestimate the species potential range. However, if occurrences from nonnative ranges were available for validating the models, this could increase the predictive power resulting in more refined predictions. Additionally, our predictions could have been improved if we adopted a hybrid approach that includes fitting the SDMs with existing ecological knowledge. Recently, development of Bayesian SDM using Gaussian process (GP) by Golding and Purse^37^, enables the user to incorporate prior ecological knowledge via a prior estimate of a model function.

Correlative SDMs (MaxEnt and GARP) are focused primarily on the realized niche and in turn, are capable of representing only a part of the fundamental niche. Additionally, when dispersal is not included into habitat projections, potential habitat and colonizable habitat can differ significantly^38^. This can lead to a great deal of uncertainty when deciding on resource allocation for FIAS control measures and further lead to huge economic losses. Our approach of addressing dispersal limitations using MigClim allows simulating FIAS spread for a set of anticipated future climate change conditions while integrating species-specific genetic traits (flight capacity, long dispersal distance, etc.). We have found suitable areas where AGM is likely to spread if it gets introduced and establishes in Vancouver. Such information can be used by managers to more finely focus eradication efforts.

Due to increasing trade, climate change, and a lack of established gypsy moth natural enemies, Canada’s forests are under acute risk of being invaded by AGM^4,39^. A recent study by Paini *et al*.^13^ found a total of 7,662 ships arriving at Canadian ports in 2005 which came to Canada directly from a port in a region where AGM is able to establish. Thus, maps produced from this study will help in providing information about the potential suitable distribution ranges of AGM for formulating effective mitigation strategies and aid in designing pest surveys and domestic quarantines. However, the maps produced should be interpreted with caution as there is no best transferable SDM for all species^40^ and predictions differ with varying modelling assumptions. Also, AGM infested ships could arrive to any other vulnerable port and not just Vancouver, thus additional simulations under multiple scenarios of dispersal at various points of entry are still needed.

## Material and Methods

In order to meet the objectives the following steps were taken, that will be described in detail below:

1. AGM occurrence records were collected from several sources.
2. Spatial datasets representing current and future climate (A1B and A2) were developed alongside spatial information on the human footprint in the area of interest.
3. Both a MaxEnt and GARP model were built to simulate the potential distribution of AGM in Asia and Canada.
4. Dispersal limited projection of future AGM distribution under A1B and A2 climate change scenarios were produced using MigClim.

**Figure 6:**
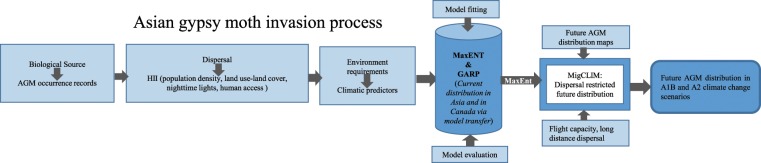
Flowchart representing the modelling flow used to model Asian gypsy moth distribution in this study.

Conceptual and analytical flowchart of present study is shown in Fig. 6.

### Occurrence data

**Figure 7:**
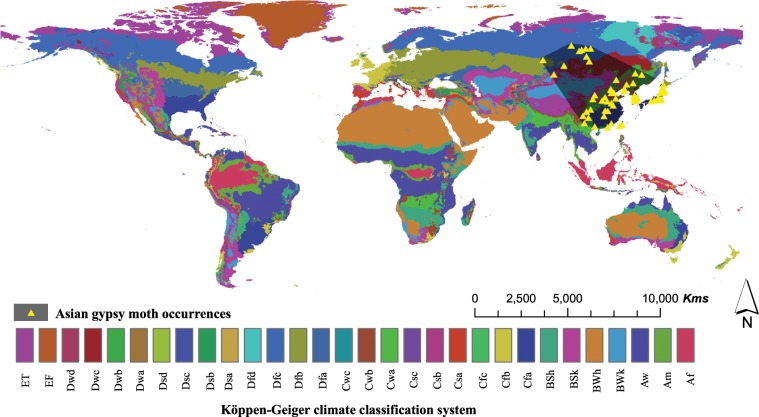
Occurrences of Asian gypsy moth. The shaded region represents the background used for creating the SDM based on a buffered minimum convex polygon. The Köppen-Geiger climate classification (vegetation-based) system^40^ was used as a background. This is done to allow assessing risk based preliminary on whether a species is found in the same climate zone as the pest risk assessment area.

To compare the performances of MaxEnt and GARP and in order to map the potential distribution of AGM, we collected presence records of AGM in its native range from various sources. The sources included (1) Global Biodiversity Information Facility database, an online database for species occurrences; (2) Records provided by the Canadian Food Inspection Agency (CFIA); and (3) Scientific articles and maps. We deleted duplicate records such that each observation falls inside a separate 20 km grid cell, leading to a total of 78 distinct occurrence records (Fig. 7). Our collected records cover a large proportion of the species’ native range and shows that AGM can survive under various climatic conditions (Table 1).

### Environmental variables

Nineteen layers of bioclimatic data on current and future climatic conditions, A1B (moderate increase in global surface warming) and A2 (higher increase in global surface warming) were obtained from CLIMOND at 20 km spatial resolution^41^. Additional data on human footprint “Human Influence Index-HII” at 1 km and vegetation based Köppen-Geiger climatic zones at 20 km were obtained from SEDAC^42^ and CLIMOND^41^ respectively. The Human Influence Index (HII) is a measure of direct human influence on terrestrial ecosystems, derived from nine global variables including land cover, population density, built-up areas, roads, navigable rivers and nighttime lights^43^. All predictor variables were resampled to 20 km resolution using a bilinear interpolation in WGS84 projection and were clipped in ArcGIS v 10.3^44^ to the native (Asia) and nonnative range (Canada) of AGM to allow model projections via SDM transfer. Further, these predictors were subjected to multicollinearity test using Pearson’s correlation coefficient (r), as the real relationship between species occurrences and environmental layers will not appear in case of heavy auto correlated variables. Therefore, we selected only one variable from each set of highly correlated variables (r > 0.7) depending on its biological relevance to the species and relative contribution to the overall model and finally selected six environmental variables: Bio1 (Annual mean temperature), Bio2 (Mean diurnal temperature range), Bio12 (Annual precipitation), Bio15 (Precipitation seasonality), HII (Human influence index) and Climatic zones for our SDM.

### Species distribution models

In this study, we used two species distribution modelling algorithms, MaxEnt and GARP to map current potential distribution of AGM. These models differ both conceptually as well as statistically and are capable of accommodating complex nonlinear functions to model the relationship between species presence and predictors^45^. Both MaxEnt and GARP were built separately using native occurrences and were later projected onto Canada to map potential suitable areas for AGM establishment.

MaxEnt is a machine learning algorithm used for describing probability distributions following the principle of maximum entropy, subject to restraints imposed by presence of species and their surrounding environment^21^. The entropy is defined by the following equation:

$$H(\hat{{\rm{\pi }}})=\sum _{{\rm{x}}\in {\rm{X}}}\,\hat{{\rm{\pi }}}({\rm{x}})\text{In}\hat{{\rm{\pi }}}({\rm{x}})$$

where π is the unknown probability distribution; $\hat{{\rm{\pi }}}$ is the approximation of π; X is a finite set; x is an individual element in set X; and ln is the natural logarithm. The entropy is nonnegative and is at most the natural log of the number of elements in X.

GARP, the Genetic Algorithm for Rule-set Prediction, works in an iterative process of rule selection, evaluation, testing and incorporating or rejecting rules to produce a heterogeneous set summarizing a species ecological requirement^46^. These sets of rules are combined in a random way to generate the potential habitat of the species restricted by the environmental conditions^47^.

### Model design and operation

MaxEnt software version 3.3.3 k was used to map potential distribution of AGM. Eighty percent of the occurrence data was portioned into ten random subsets and using in the k-fold cross validation function of MaxEnt. This was done to evaluate the average behavior of the model^26^. 10,000 pseudo-absences (PAs) were generated for model training and evaluation within an area defined by a minimum sized convex polygon encompassing occurrences in the native range using SDM toolbox^48^ (Fig. 7). The output predictions were based on default parameter values (0.01 convergence limit, and 1,000 maximum iterations). In order to produce simple models with smooth fitted functions we used only hinge features^20^. We optimized the multiplier value following an approach by Padalia *et al*.^18^. Jackknife resampling was used to identify those variables that contributed most to the model. The method provides systematic resampling and leads to improved estimates of the sample parameter and a lower sampling bias^49^. The mean values of relative contribution of the environmental variables were calculated from 10 MaxEnt modelling runs. The model runs were performed using cross-validation function in MaxEnt. Additionally, the fade by clamping function was used to limit extrapolations beyond the environmental range of the training data^50^.

We used the “openModeller” software package^51^ to implement GARP as our second SDM. The “best subset” selection procedure was used using the new openModeller implementation with default parameters. The rule sets were projected onto environment layers of Asia and Canada to generate predictions of AGM distributions. The outputs of MaxEnt and GARP with habitat suitability values ranging from 0 (unsuitable) to 1 (optimal habitat) was recorded for both native and nonnative range. Habitat suitability for nonnative range (Canada) was further classified as very low (0–0.25), low (0.25–0.50), medium (0.50–0.75) and high (0.75–1.00) for easy interpretation and comparison purposes. The cut off threshold for species presence and absence was obtained from maximizing test sensitivity and specificity^32^ and was used for the suitability classification. Presence records of European gypsy moth were also overlaid on the classified map to ensure the reliability of used classification.

### Model evaluation

Model evaluation was performed using the remaining 20% of the presence data for AGM in the native range. Area under the curve (AUC) of the receiver operating characteristic, partial AUC ratio (pAUC), and sensitivity (fraction of correctly predicted presences) values were calculated using the above independent dataset. AUC provides a single measure of model performance independent of any particular choice of threshold. The AUC measures model performance that ranges from 0 to 1. A model performs well when the AUC is large. Usually, AUC values of >0.9 indicate high accuracy, values between 0.70 and 0.9 indicate good accuracy, and values between 0.5 and 0.7 indicate low accuracy^52^. In the past, authors have found the AUC test inappropriate for presence only models^53^ since it assigns similar weight to both commission and omission errors. Therefore we also used partial AUC (pAUC) as a second evaluation method. Here, the region of Receiver Operating Characteristic (ROC) space, where the omission error is less than the user defined variable (E), is considered for calculating partial AUC^54^. A pAUC value of >1.0 shows better performing model.

We extracted the same number of PAs as testing presences in order to calculate the evaluation scores. PAs were extracted in the same spatial range as the presences. Partial AUC ratio and sensitivity were calculated at 0% omission rate. AUC and pAUC scores were calculated using “ROCR” R package^55^ and “NicheToolBox^56^” respectively.

### Dispersal mapping

In order to include AGM specific dispersal constraints into projections of its potential distributions under climate change, we used MigClim^57^. MigClim is a function library built in R software^58^ that allows implementation of species dispersal limitations in SDM predictions under climate change conditions. MigClim is a cellular automaton model so cells are the measured units and here each cell corresponds to 20 km square pixel. Since MigClim does not generate habitat suitability maps itself, we used MaxEnt to generate the required inputs. Future AGM distribution maps for the year 2030 and 2050 were produced for climate change scenarios A1B and A2 using MaxEnt. These maps were used as an input along with an initial distribution map of AGM. We assumed the initial infestation point was Vancouver port since the species was able to reproduce there in the 1990’s resulting in an eradication. We chose a reclassification threshold of 0.5 along with a dispersal kernel of 1 and 0.5, since females fly on average less than 1 km (maximum range of 20–40 km). AGM long distance dispersal frequency was set to 0.1 and min-max distance range as 100 (5 cells) and 200 km (10 cells) respectively^59^. Propagule production potential was set to 1. We had two environmental change steps (2030 and 2050) where each environmental change step had 20 dispersal steps. Accordingly the total number of dispersal steps simulated was equal to [envChgSteps] × [dispSteps], here 40, which corresponds to 40 years from 2010 to 2050. The simulations were repeated for 10 year timeframes producing dispersal limited future distribution maps of AGM from 2010 to 2050 under A1B and A2 climate change scenarios.

### Disclaimer

The use of trade, firm, or corporation names in this publication is for the information and convenience of the reader. Such use does not constitute an official endorsement or approval by the U.S. Department of Agriculture or the Forest Service of any product or service to the exclusion of others that may be suitable.
